# Summary of best evidence for non-pharmacological management of sleep disturbances in patients with mild cognitive impairment or early dementia: an integrative review

**DOI:** 10.3389/fmed.2026.1913461

**Published:** 2026-09-09

**Authors:** Qi Xie, Jing Shi, Yinning Guo, Mingyue Hu, Xi Chen, Yishi Chen, Yanhong Dong, Zhihong Wei, Hui Feng

**Affiliations:** 1 Xiangya Nursing School, Central South University, Changsha, China; 2 Alice Lee Centre for Nursing Studies, Yong Loo Lin School of Medicine, National University of Singapore, Singapore, Singapore; 3 Intensive Care Unit, The Second Hospital & Clinical Medical School, Lanzhou University, Lanzhou, Gansu, China; 4 School of Nursing, Nanjing Medical University, Nanjing, China; 5 Department of Medicine, Yong Loo Lin School of Medicine, National University of Singapore, Singapore, Singapore; 6 Outpatient Department, The Second Hospital & Clinical Medical School, Lanzhou University, Lanzhou, Gansu, China; 7 Oceanwide Health Management Institute, Central South University, Changsha, China; 8 National Clinical Research Center for Geriatric Disorders, Xiangya Hospital, Central South University, Changsha, China

**Keywords:** best evidence, cognitive impairment, non-pharmacological interventions, prevention and management, sleep disorders

## Abstract

**Aim:**

To consolidate the best evidence on non-pharmacological management of sleep disturbances in patients with mild cognitive impairment (MCI) or early dementia, providing a basis for clinical and community healthcare professionals to develop evidence-based sleep care plans.

**Methods:**

Following the “6S” evidence resource model, evidence was retrieved using a top-down approach, prioritizing higher-level synthesized evidence. Eligible evidence types included clinical practice guidelines, expert consensus documents, best practice reports, clinical decision-support documents, evidence summaries, and systematic reviews. A comprehensive search was conducted from database inception to August 2025 across point-of-care evidence resources, guideline repositories, professional organization websites, and bibliographic databases.

**Results:**

Twenty-two publications were included: 2 guidelines, 3 best practice reports, 4 expert consensus documents, 4 clinical decision documents and 9 systematic reviews. From these, 44 best evidence items were identified across 11 themes: risk factors, sleep screening, sleep assessment, sleep hygiene, cognitive behavioral therapy, light intervention, exercise interventions, multimodal interventions, other interventions, sleep monitoring, and health education and social support.

**Conclusion:**

This study summarizes the best evidence for non-pharmacological management of sleep disorders in people with MCI and early dementia across 11 domains. These pieces of evidence can provide guidance for clinical or community healthcare professionals in formulating and implementing sleep care plans for this population, and may help improve sleep outcomes, quality of life, and caregiver well-being. In clinical practice, professionals should make context-specific judgments and fully consider patient preferences and resource constraints when selecting and applying the evidence.

**Systematic Review Registration:**

http://ebn.nursing.fudan.edu.cn/home, identifier: ES202610453.

## 1 Introduction

Sleep disturbances are among the most prevalent and debilitating non-cognitive symptoms in patients with mild cognitive impairment (MCI) and early dementia (1). The prevalence of sleep disorders in older adults with MCI and early dementia has been reported to range from 57.52% to 70% (2–4). These disorders primarily manifest as insomnia, excessive daytime sleepiness, sleep-disordered breathing, parasomnias, restless legs syndrome, and circadian rhythm sleep-wake disorders (5). They exhibit a bidirectional relationship with the underlying neuropathology, potentially accelerating cognitive decline, exacerbating neuropsychiatric symptoms such as agitation and depression (6), and significantly impairing the quality of life for both patients and their caregivers (7). Early identification and management of sleep problems may therefore improve quality of life and potentially reduce long-term cognitive risk in older adults with MCI and early dementia.

Sleep interventions for older adults with MCI and early dementia primarily include pharmacological and non-pharmacological interventions (NPIs) (3). Although pharmacological treatments may provide relatively rapid symptom relief, the use of pharmacological agents in older adults with cognitive impairment is associated with important safety concerns, including an increased risk of cerebrovascular events, sudden death, falls, and other adverse drug events (8). Consequently, clinical guidelines generally recommend NPIs as the preferred first-line management strategy (9). Research indicates that NPIs such as environmental modifications, light therapy, acupressure, and multisensory stimulation may be effective in managing sleep problems in patients with dementia (10). However, most existing studies have focused on individual interventions, and findings regarding their effectiveness remain inconsistent (3, 11, 12).

Comprehensive non-pharmacological sleep management should include sleep assessment, selection and implementation of appropriate interventions, follow-up, ongoing monitoring, and other relevant components. Nevertheless, the available evidence is largely dispersed across general dementia care guidelines, recommendations for specific types of sleep disturbances, systematic reviews, and other clinical evidence resources (13, 14). Evidence specifically addressing sleep management in people with MCI or early dementia remains fragmented and has not yet been systematically integrated into a practical, evidence-based framework for clinical guidance. Existing studies also indicate that healthcare professionals have limited knowledge and confidence in identifying, assessing, and managing sleep disturbances in people with MCI or early dementia (15, 16). In the absence of systematic guidance and standardized management pathways, clinical practice is often shaped by self-directed learning, personal experience, and local routines, resulting in variation and inconsistency in sleep assessment and management across care settings (16, 17). Therefore, it is essential to provide healthcare professionals with evidence-based recommendations to support the development of scientifically sound, effective, and accurate strategies for the prevention and management of sleep disturbances in this population.

Best evidence summaries are an important component of evidence-based practice. They aim to identify, appraise, and synthesize the best available evidence from various sources, such as clinical guidelines, expert consensus, systematic reviews, and evidence summaries, to address a specific clinical question and translate the evidence into clear and actionable recommendations for clinical practice (18). Therefore, this study aimed to synthesize the best available evidence on the non-pharmacological management of sleep disturbances in individuals with MCI and early dementia. The resulting evidence summary was intended to support early identification, clinical decision-making, and appropriate sleep management in this population.

## 2 Methods

Currently, there is a lack of standardized reporting guidelines for evidence summaries (19). The Fudan University Centre for Evidence-based Nursing has developed a reporting framework based on the JBI methods for generating evidence summaries. This framework includes problem establishment, evidence retrieval, screening and appraisal, evidence extraction, synthesis and grading, and the formulation of practical recommendations (20). Applicable PRISMA 2020 items were also applied to improve the transparency of evidence identification, screening, and selection. This study has been registered and approved, following the reporting standard established by the Fudan University Centre for Evidence-based Nursing, under registration number ES202610453. (http://ebn.nursing.fudan.edu.cn/home).

### 2.1 Problem establishment

Specific issues in constructing evidence summary reports using the PIPOST model proposed by the Evidence-based Nursing Research Centre of Fudan University in this study (21). (1) P (Population): older adults with MCI or early-stage dementia. (2) I (Intervention): included non-pharmacological approaches to sleep management, such as sleep screening and assessment, implementation of sleep interventions, follow-up, and ongoing monitoring. (3) P (Professional): Clinical practitioners. (4) O (Outcome) was indicators related to improving sleep quality. (5) S (Setting): hospitals, long-term care facilities, communities and homes. (6) T (Type of Evidence): guidelines, expert consensus statements, clinical decisions, systematic reviews, best practice reports, and evidence summaries.

### 2.2 Evidence retrieval

The top-down search strategy was based on the “6S” evidence resource model (22). The model organizes evidence resources into six hierarchical levels: Systems, Summaries, Synopses of Syntheses, Syntheses, Synopses of Single Studies, and Single Studies (23). Evidence retrieval generally begins at the highest applicable level and proceeds downward as needed. In this study, the 6S model was used to guide the priority and sequence of evidence retrieval within the predefined eligible evidence types. Accordingly, priority was given to more highly synthesized and pre-appraised evidence, including clinical decisions, clinical guidelines, best practice reports, and evidence summaries, followed by systematic reviews and expert consensus. Guided by this model, we systematically searched the following sources: (1) point-of-care evidence resources: BMJ Best Practice, UpToDate, and JBI Best Practice; (2) guideline repositories: the National Institute for Health and Care Excellence (NICE), the National Guideline Clearinghouse, the Guidelines International Network, the Scottish Intercollegiate Guidelines Network (SIGN), the Registered Nurses' Association of Ontario (RNAO), and the Chinese Medlive Guideline Network; (3) professional associations and specialty societies: Alzheimer's Disease International (ADI), the American Psychological Association (APA), the American Geriatrics Society (AGS), the European Academy of Neurology (EAN), and the American Academy of Sleep Medicine (AASM); (4) bibliographic databases: Cochrane Library, PubMed, CINAHL, Embase, Web of Science, China Biology Medicine (CBM), China National Knowledge Infrastructure (CNKI), and Wanfang Database.

The search strategy was developed with the assistance of an experienced librarian. Because web-based clinical resources, guideline repositories, and professional society websites generally provide limited search functionality and may index relevant guidance under broader topic headings, source-specific search approaches were used. Searches were conducted using individual terms and simplified combinations related to cognitive impairment (e.g., “cognitive dysfunction,” “cognitive impairment,” and “cognitive decline”) and sleep disturbances (e.g., “sleep disorder,” “sleep problems,” and “sleep disturbances”), according to the search functionality of each resource. When combined searches yielded few or no relevant results, individual terms were searched iteratively. All potentially relevant documents were screened against the prespecified eligibility criteria. For bibliographic databases, the search strategies combined Medical Subject Headings (MeSH) with free-text terms. The literature search formula consists of three parts: the first of which was cognitive dysfunction or cognitive impairment–related terms, the second was sleep/sleep disorder–related terms and the third was a restriction on the type of literature to be searched. The search timeframe is from database inception to August 2025. No study registries or additional grey literature sources were systematically searched. No published search filters were used. The reference lists of included articles and other relevant systematic reviews were examined. Authors were contacted when necessary to clarify eligibility or obtain missing information. Detailed search strategies are provided in Supplementary Table S1.

### 2.3 Inclusion and exclusion criteria for evidence

The inclusion criteria were as follows: (1) participants were adults aged ≥60 years with MCI or early dementia. Sources involving broader populations were eligible only when findings or recommendations specifically applicable to the target population could be clearly identified and extracted. (2) research topics were NPIs for sleep disturbances in this population, requiring clear recommendations or specific methods for managing such sleep issues without medication; (3) eligible publication types comprised clinical guidelines, expert consensus, clinical decision, systematic reviews, best practice reports, and evidence summaries; (4) The publication language was either Chinese or English; No lower publication-date limit was imposed.

Exclusion criteria encompassed: (1) literature for which full text was unavailable, duplicate publications, or incomplete information; (2) document types such as conference abstracts, translated versions of international guidelines, guideline interpretations or drafts, and research protocols; (3) the literature failed to meet the predefined methodological quality appraisal criteria.

### 2.4 Literature screening and evidence extraction

Two researchers systematically trained in evidence-based methodology independently screened the retrieved literature according to the predefined inclusion and exclusion criteria. The screening procedure consisted of three phases: Firstly, duplicate records were removed using EndNote software. Subsequently, titles, abstracts, and keywords were reviewed to exclude obviously irrelevant publications. Finally, the full texts of the remaining articles were thoroughly examined to identify eligible studies. Any discrepancies during the screening process were resolved through discussion between the two reviewers. If consensus could not be reached, a third evidence-based nursing expert was consulted for arbitration. A standardized data extraction form was used to systematically collect key information, including authors/institute, publication year, literature source, evidence type, the literature theme, evidence content, and support reference. Two researchers independently completed evidence extraction. Disagreements were resolved by re-examining the source documents with focused verification. Final unresolved disagreements were referred to a third researcher for adjudication.

### 2.5 Literature quality evaluation criteria

Different assessment tools were used according to literature types. AGREE II for clinical practice guidelines, AMSTAR 2 for systematic reviews, the JBI checklist for expert consensus documents, and CASE for best practice reports, clinical decisions, and evidence summaries. The Appraisal of Guidelines for Research and Evaluation II (AGREE II) instrument comprises 23 items across six domains: scope and purpose, stakeholder involvement, rigor of development, clarity of presentation, applicability, and editorial independence (24, 25). Each item is rated on a seven-point scale ranging from 1 (strongly disagree) to 7 (strongly agree). Standardized scores in each domain = (obtained score−minimum possible score)/(maximum possible score−minimum possible score)  ×  100% (24). To determine the overall quality of each included guideline, we applied the following *a priori* cut-off criteria, based on published methodological recommendations and previous AGREE II appraisal studies (26–28): if 6 domains score ≥60%, it is recommended as a grade A; if ≥3 domains score ≥30% with some domains with scores <60%, it is recommended as a grade B after modification and improvement; and ≥ 3 domains with scores <30% are excluded as grade C.

The Assessment of Multiple Systematic Reviews 2 (AMSTAR 2) was used to evaluate systematic reviews in this study (29). AMSTAR 2 assesses 16 items, including protocol registration, literature search, study selection, risk-of-bias assessment, synthesis methods, and publication bias. Of the 16 items, seven were considered critical domains (items 2, 4, 7, 9, 11, 13, and 15). Literature with no or only 1 non-key entry nonconformity is considered high quality; literature with more than 1 non-key entry nonconformity is considered medium quality; literature with only 1 key entry nonconformity is considered low quality; literature with more than 1 key entry nonconformity is considered very low quality (29). Reviews rated as critically low confidence were excluded.

The expert consensus was evaluated using the JBI Critical Appraisal Checklist for Text and Opinion Papers (30, 31). The checklist evaluates the credibility and support of expert opinion. Each item was rated as “Yes”, “No”, “Unclear” or “Not applicable”, followed by an overall appraisal decision of “Include”, “Exclude” or “Seek further information”. Sources receiving an overall decision of “Include” were retained. Sources requiring further information were re-examined, and the final decision was made through reviewer discussion (32).

Best practice reports, clinical decisions documents and evidence summaries were evaluated using the Critical Appraisal for Summaries of Evidence (CASE) tool (33, 34). This tool covers 4 aspects (topic, methods, content, and applicability) with a total of 10 items, and each item has 3 evaluation criteria: “Yes,” “No,” and “Partially Yes.” A literature was excluded if more than 5 items were rated “No,” or if the proportion of items rated “Partially Yes” was ≥ 60% (33, 35).

Methodological quality appraisal was conducted independently by two reviewers trained in evidence-based methodology. Disagreements were resolved through discussion or, when necessary, consultation with a third reviewer experienced in evidence-based research.

### 2.6 Evidence synthesis and grading

After the evidence extraction was completed, the evidence was classified and integrated. Principles for evidence synthesis were as follows (36, 37): (1) the included sources were not treated as equivalent during synthesis; Clinical practice guidelines and systematic reviews were generally used as principal sources for recommendation content, whereas expert consensus documents provided supportive evidence and clinical decision-support resources and evidence summaries were used primarily to supplement implementation details. This role-based prioritization was not applied mechanically; methodological quality, population directness, currency, and clinical applicability were also considered; (2) for consistent evidence, concise and clear recommendations were prioritized; (3) for complementary evidence, items were logically combined to form integrated evidence statements; (4) for conflicting evidence, priority was given to evidence that was methodologically stronger, more recent, and more locally applicable. After evidence integration, the draft evidence statements were organized using inductive content-based categorization (38). Each statement was treated as the unit of analysis and independently grouped by two reviewers according to similarities in its primary clinical purpose and content. The resulting categories were iteratively reviewed, merged, and refined through team discussion until consensus was reached.

Evidence grading and recommendation strength were determined using the JBI evidence level and recommendation grading approach (39, 40). Evidence-level classification was conducted independently of the methodological quality appraisal. Evidence levels were determined according to the JBI Levels of Evidence (2014) (40). For each evidence item, the appropriate JBI hierarchy was first selected according to the type of clinical question addressed (e.g., effectiveness, diagnosis, or prognosis). The specific evidence level was then assigned according to the study design of the supporting evidence. For effectiveness-related evidence, Level 1 represents experimental designs, Level 2 quasi-experimental designs, Level 3 observational-analytic designs, Level 4 observational-descriptive designs, and Level 5 expert opinion and bench research. Using the JBI evidence rank system, recommendations as A (strong) or B (weak) based on assessments of feasibility, appropriateness, clinical meaningfulness, and effectiveness (39, 41). The resulting evidence grading and recommendation strength were discussed, evaluated, and refined during an expert panel meeting until consensus was reached. The panel comprised six experts: two evidence-based methodology specialists and four clinical experts (sleep medicine specialist, neurology specialist, geriatric nursing specialists and community care specialists).

## 3 Results

### 3.1 General characteristics of the included literature

**Figure 1:**
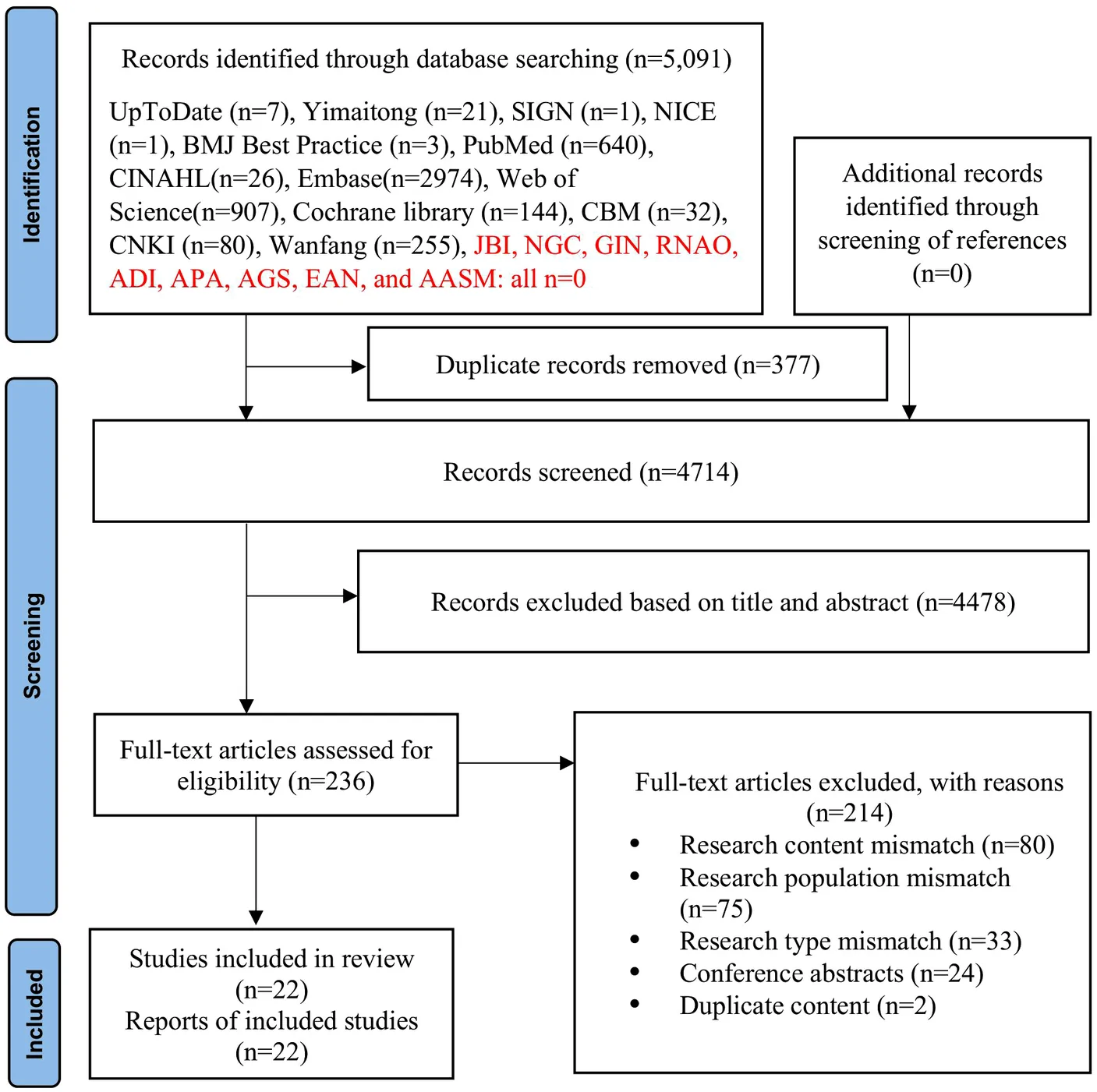
Article selection flow diagram (modelled as per PRISMA).

A preliminary search identified 5091 relevant publications. After duplicate removal and sequential screening of titles, abstracts, and full texts, 22 evidence sources were ultimately included. These comprised 2 guidelines (42, 43), 3 best practice reports (44–46), 4 expert consensus (47–50), 4 clinical decisions (51–54), and 9 systematic reviews (11, 14, 55–61). The literature selection process is illustrated in Figure 1, while the basic characteristics of the included publications are detailed in Table 1.

**Table 1:** Characteristics of included literature (*n* = 22).

Included literature	Year of publication	Literature sources	Type of literature	The literature theme
Larson, EB	2024	UpToDate	Clinical decision	Risk factors for cognitive decline and dementia
Feinsilver, SH.	2024	UpToDate	Clinical decision	Causes of impaired sleep including sleep apnea in older adults
Ofer Jacobowitz	2024	BMJ Best Practice	Best Practice	Obstructive sleep apnoea in adults
Joshua Hyong-Jin Cho	2025	BMJ Best Practice	Best Practice	Insomnia
BMJ	2024	BMJ Best Practice	Best Practice	Parasomnias in adults
B.Guarnieri • M	2014	PubMed	guideline	Recommendations of the Sleep Study Group of the Italian Dementia Research Association (SINDem) on clinical assessment and management of sleep disorders in individuals with mild cognitive impairment and dementia: a clinical review
Manuel Montero-Odasso	2020	PubMed	Expert consensus	CCCDTD5 recommendations on early non cognitive markers of dementia: A Canadian consensus
Petersen, RC	2024	UpToDate	Clinical decision	Mild cognitive impairment: Epidemiology, pathology, and clinical assessment
Petersen, RC	2024	UpToDate	Clinical decision	Mild cognitive impairment: Prognosis and treatment
Chinese Sleep Research Society	2024	National Medical Journal of China	Expert consensus	Chinese Expert Consensus on the Diagnosis and Treatment of Insomnia Disorder in Primary Care Settings
Chinese Sleep Research Society	2024	Wanfang	Expert consensus	Chinese Expert Consensus on Digital Therapy for Insomnia
R Robert Auger	2015	PubMed	guideline	Clinical Practice Guideline for the Treatment of Intrinsic Circadian Rhythm Sleep-Wake Disorders: Advanced Sleep-Wake Phase Disorder (ASWPD), Delayed Sleep-Wake Phase Disorder (DSWPD), Non-24-Hour Sleep-Wake Rhythm Disorder (N24SWD), and Irregular Sleep-Wake Rhythm Disorder (ISWRD). An Update for 2015: An American Academy of Sleep Medicine Clinical Practice Guideline
Jeff W Jin	2021	PubMed	Systematic review	Cognitive Behavioral Therapy for Mood and Insomnia in Persons With Dementia A Systematic Review
Gao et al.	2024	Wanfang	Systematic review	The Impact of Mindfulness-Based Intervention on Sleep Quality in Patients with Mild Cognitive Impairment: A Meta-Analysis
Huei-Ling Chiu	2017	PubMed	Systematic review	Effectiveness of Light Therapy in Cognitively Impaired Persons: A Metaanalysis of Randomized Controlled Trials
Junlei Zhang	2024	PubMed	Systematic review	Comparative efficacy of various exercise interventions on sleep in patients with cognitive impairment: a systematic review and meta-analysis
Ahn, J	2023	PubMed	Systematic review	Effects of aerobic exercise on global cognitive function and sleep in older adults with mild cognitive impairment: A systematic review and meta-analysis
Chen, L	2024	PubMed	Systematic review	Exercise effects on neuropsychiatric symptoms and quality of life in mild cognitive impairment: a systematic review and meta-analysis.
Liu, X	2023	PubMed	Systematic review	The effectiveness of exercise on global cognitive function, balance, depression symptoms, and sleep quality in patients with mild cognitive impairment: A systematic review and meta-analysis
Rónán O'Caoimh	2019	PubMed	Systematic review	Non-pharmacological treatments for sleep disturbance in mild cognitive impairment and dementia: A systematic review and meta-analysis
Wang et al.	2025	Wanfang	Expert consensus	Chinese Expert Consensus on Diversified Rehabilitation Interventions for Alzheimer's Disease (2025)
Jonathan Blackman	2020	PubMed	Systematic review	Pharmacological and non-pharmacological interventions to enhance sleep in mild cognitive impairment and mild Alzheimer's disease: A systematic review

### 3.2 Quality evaluation results of the included literature

#### 3.2.1 Quality evaluation results of the guidelines

Among the 2 included guidelines, one was classified as Grade A, with standardized scores of ≥60% in all six AGREE II domains, whereas the other was classified as Grade B. Detailed standardized domain scores and overall appraisal results are presented in Supplementary Table S2.

#### 3.2.2 Quality evaluation results of the best practice reports and clinical decisions

All three best practice reports and four clinical decisions met the predefined CASE appraisal criteria and were retained for inclusion (Supplementary Table S3).

#### 3.2.3 Quality evaluation results of expert consensuses

Four expert consensus articles were independently evaluated by two reviewers using the JBI Critical Appraisal Checklist for Text and Opinion Papers. All received an overall appraisal decision of “Include” and were retained in the evidence synthesis. Detailed quality assessment results are presented in Supplementary Table S4.

#### 3.2.4 Quality evaluation results of systematic reviews

Five studies were rated as high quality, one as medium, and three as low. Detailed quality assessment results are presented in Supplementary Table S5.

### 3.3 Summary and description of evidence

From the 22 included sources, 61 preliminary evidence statements were extracted. After consolidation of consistent and complementary content, 44 best evidence statements remained. These were organized into 11 inductively derived themes: risk factors, sleep screening, sleep assessment, sleep hygiene, cognitive behavioral therapy (CBT), light intervention, exercise intervention, multimodal intervention, other interventions, sleep monitoring, health education and social support (Table 2). Each final evidence statement was subsequently assigned an evidence level and recommendation grade using the JBI framework.

**Table 2:** Summary of best evidence for Non-pharmacological interventions of sleep disorders in patients with MCI and early dementia.

Evidence items		Evidence content	Level of evidence	Recommended level
Risk factors	Screening prioritization	Prioritize sleep disorder screening in MCI/early dementia patients with strong risk factors for insomnia/OSA (46, 51, 52).	3a	A
	Strong risk factors — Insomnia/OSA	Recommend prioritizing insomnia screening in individuals with: women, older age, chronic diseases, psychiatric disorders, substance abuse, and traumatic brain injury. (45)	3e	A
		Recommend prioritizing obstructive sleep apnea (OSA) screening in individuals with: obesity, male, postmenopausal status, large neck circumference, upper-airway anatomical abnormalities, family history of OSA, chronic snoring, and smoking. (46)	3c	A
	Weak risk factors — Insomnia/OSA	Consider insomnia screening in individuals with: use of certain medications (e.g., SSRIs, bronchodilators, decongestants) and sedentary behavior. (45)	4a	B
		Consider OSA screening in individuals with: nasal congestion, testosterone use (men), alcohol consumption, secondhand smoke exposure, and chronic opioid use. (46)	4c	B
Sleep screening	Screening Object	Incorporate sleep screening into the evaluation of older adults with cognitive complaints or those undergoing cognitive assessment, in both primary care and memory clinics (45, 47, 53, 54).	5b	A
	Screening Content	Screening should cover sleep duration, insomnia symptoms, daytime sleepiness, napping habits, and behaviors suggestive of REM sleep behavior disorder (RBD) (45, 47).	1a	A
	Screening Tool	Use validated questionnaires for initial screening, such as PSQI/ISI/AIS (sleep quality/insomnia), ESS or SSS (daytime sleepiness), MEQ (chronotype), STOP-Bang or Berlin Questionnaire (OSA risk), and RLSQ (RLS symptoms) (45, 49, 50).	3c	A
	Screening condition for referral	If screening suggests clinically significant sleep disturbance or high-risk features, proceed to structured assessment and consider referral for disorder-specific evaluation (e.g., suspected OSA) (45, 47).	5b	A
Sleep assessment	Assessment Principle	Diagnosis is primarily based on clinical interview; identify etiology/contributing factors and comorbidities, using caregiver input when needed (43, 45).	1b	A
		In long-term care residents, assess excessive daytime sleepiness (EDS) (43).	1b	A
	Assessment Tool	Recommend brief cognitive screening (e.g., MoCA or MMSE) during assessment (45).	1b	A
		Recommend a standard sleep diary to document bedtime, sleep onset latency, total sleep time, nocturnal awakenings, morning wake time/rise time, and subjective sleep quality (45).	1a	A
		For suspected circadian rhythm disturbance, maintain a sleep diary for ≥7 days and consider actigraphy to objectively document sleep–wake patterns (43, 50).	1a	B
		Recommend polysomnography (PSG) for diagnostic confirmation when OSA is suspected; use video-PSG (vPSG) when RBD is suspected (43, 46).	5b	A
		If screening suggests RLS, assess severity and impact using a validated scale (49).	5b	A
Sleep hygiene	Principle	Recommend sleep hygiene education as the foundational component of non-pharmacological management for sleep problems in individuals with MCI/early dementia; it should generally be combined with other interventions rather than used as a stand-alone treatment (42, 43, 45).	1a	B
	Core components	Recommend a regular sleep–wake schedule, with consistent bedtimes and wake times, including weekends (44, 45).	5b	B
		Optimize the sleep environment (dark, quiet, comfortable temperature/humidity, and comfortable bed/bedding) (45). Avoid sleep-disrupting substances and screens before bedtime, including alcohol, caffeine, nicotine, and blue-light–emitting devices (e.g., smartphones/computers) (44, 45).	5b	B
		Avoid prolonged daytime napping (or limit naps when necessary) (44, 45), and reduce pre-sleep stress and cognitive arousal, avoiding work-related rumination close to bedtime (44).	5b	B
CBT	Intervention Role	Recommend CBT-I as the first-line treatment for chronic insomnia (45, 58).	1a	A
	Intervention Setting	CBT-I can be delivered in primary care and community settings (45).	1a	A
	Core components	Include stimulus control, sleep restriction, cognitive restructuring, and relaxation training (45).	5b	B
	Intervention Delivery	Recommend dCBT-I to improve accessibility when face-to-face CBT-I is limited (45, 50).	1b	A
Light intervention	Intervention Role	Recommend bright light therapy to stabilize the sleep–wake rhythm and improve nocturnal sleep quality, particularly in cognitively impaired individuals (43, 49).	5b	A
	Intervention Dose	Use morning bright light exposure at 2,500–10,000 lux for 30–45 min daily, with consistent application (49, 57).	5b	A
	Intervention Timing	For phase advance or early-morning awakening, recommend late-afternoon or evening bright light to delay circadian timing and improve sleep maintenance (43). For phase delay, recommend morning bright light exposure to advance circadian timing and facilitate earlier sleep onset (43, 49).	5b	B
Exercise intervention	Intervention Principle	Recommend regular exercise for individuals with MCI/early dementia to support sleep, tailored to preference and physical capacity, and maintained for ≥3–6 months (42, 49, 60).	5b	A
	Intervention Type	Use aerobic exercise (e.g., brisk walking, cycling, swimming) ≥ 45 min/session, 3 sessions/week, for ≥4–6 months (42). Use resistance training (e.g., bands, dumbbells, bodyweight) ∼60 min/session, 2 sessions/week, for ≥6 months (42). Recommend multicomponent exercise (aerobic + resistance + balance/coordination) 30–60 min/session, 3–4 sessions/week, for ≥4 months (42). Recommend traditional or mind–body exercise (e.g., Baduanjin, Tai Chi, yoga) 30–60 min/session, 3–5 sessions/week, for ≥3–6 months (42, 49). Recommend exergaming as an alternative or supplement 45–60 min/session, 2–3 sessions/week, for ≥3 months (42).	5b	B
Multimodal intervention	Intervention Components	An individualized multicomponent intervention that may include daytime bright light exposure, evening light restriction, daytime physical/social activities, a regular sleep–wake schedule, and core CBT-I components, may improve sleep efficiency and subjective sleep quality (14, 42, 43).	1a	A
Other interventions	Weight reduction (OSA risk reduction)	Recommend weight reduction for overweight/obese individuals with OSA; consider bariatric surgery when clinically appropriate (44).	5b	A
	Environment safety (RBD)	Recommend sleep environment safety modifications for suspected/confirmed RBD to reduce injury risk (e.g., remove hazardous objects; add protective barriers around the bed) (43).	5b	A
	Mindfulness-based program	Consider a mindfulness-based program (6–8 weeks, weekly 60–90 min sessions) as an adjunct or alternative to CBT-I to improve subjective sleep quality in MCI (61).	1a	A
	Horticultural therapy	Consider horticultural therapy 1–2 sessions/week, 1–2 h/session, for ≥6 weeks (42, 48).	1d	B
	Multisensory stimulation	Consider multisensory stimulation 2 sessions/week, 30–60 min/session, for 4–8 weeks (42).	5b	A
	Aromatherapy	Consider aromatherapy 5 sessions/week, 15 min/session, for 4 weeks (42).	5b	A
	Neuromodulation	Consider neuromodulation (e.g., rTMS/tES) only as an adjunct/investigational option in specialized settings after contraindications are excluded and standardized protocols are used (11, 48).	1d	A
Sleep monitoring	Follow-up frequency	Recommend follow-up within 2–4 weeks for acute insomnia (45).	1b	A
		Recommend regular follow-up for chronic insomnia or CRSD to monitor symptoms, treatment response, and adherence (45).	1b	A
		For RBD, assess at 1 month after initiating treatment, then every 3–6 months to monitor efficacy and adherence (44, 45).	5b	A
Health education and social support	Practical materials	Provide concise practical manuals (sleep hygiene basics, regular sleep–wake routines, daytime activity/light routines in care settings, RBD safety checklist, and sleep diary instructions) (42–44, 49).	5b	A
	Caregiver involvement	Encourage bed partners/caregivers to participate in assessment and intervention delivery to support adherence and safety (42–44, 49).	5b	A
	Implementation in long-term care/community	In long-term care/community settings, implement routine non-pharmacological practices (regular sleep–wake schedules, structured daytime light exposure and activities, reduced nighttime noise/light) with caregiver training and team-based support (42, 43).	1a	A
	Digital and self-help resources	Consider digital and self-help resources to improve access and adherence, including dCBT-I where appropriate, non-diagnostic self-monitoring, and adjunct relaxation-based strategies (45, 49, 50).	1b	A

## 4 Discussion

Sleep disturbances are modifiable and are associated with the severity of cognitive impairment. Early identification and timely NPIs can improve sleep problems in individuals with MCI and early dementia, potentially enhancing quality of life and alleviating caregiver burden (62, 63). This review systematically retrieved and appraised 22 evidence sources on NPIs for sleep disorders in this population, graded evidence using the JBI evidence pre-grading system, and synthesized 44 best-evidence recommendations across 11 domains to support clinical nursing practice.

### 4.1 Assessment of high-risk factors

Sleep disturbances in individuals with MCI and early dementia likely reflect the interaction of neurodegeneration-related changes in sleep–wake regulation with demographic, clinical, and behavioral factors (64, 65). Routinely obtaining a sleep history during cognitive assessment—including sleep duration, insomnia symptoms, daytime sleepiness, and napping—may help identify possible sleep disturbances and guide further evaluation (47).

Non-modifiable factors associated with insomnia include sex and age. Female sex has frequently been associated with a higher risk of insomnia, potentially related to a greater burden of affective symptoms and hormonal changes across the menopausal transition (66, 67). The association between older age and insomnia is also commonly observed, but remains difficult to interpret because it may reflect both age-related changes in sleep architecture and the accumulating burden of comorbid conditions and polypharmacy; differences in how studies measure sleep and control confounding likely contribute to inconsistent estimates (68, 69). Potentially modifiable clinical contributors include chronic medical illness, chronic pain, anxiety, and depression. Chronic illness and pain may promote sleep fragmentation through persistent symptoms, inflammation, and autonomic activation, whereas anxiety and depression are closely linked to insomnia through cognitive and physiological hyperarousal and rumination (70, 71).

Risk factors for obstructive sleep apnea (OSA) should also be considered separately. Obesity is the most established modifiable risk factor, largely because regional adiposity increases upper-airway collapsibility and ventilatory load (72). Higher risk among men and postmenopausal women may relate to sex- and hormone-associated differences in fat distribution and upper-airway stability (73, 74). Overall, assessment of both modifiable and non-modifiable factors may help identify individuals with MCI or early dementia who require more focused screening and individualized management.

### 4.2 Implementation of integrated assessment and tailored interventions

To translate the synthesized evidence into practice, sleep management in individuals with MCI or early dementia may be organized around a structured pathway that links assessment, identification of the predominant sleep problem and its contributing factors, and selection of targeted NPIs. Because cognitive impairment may limit symptom reporting, assessment should integrate clinical interviews and caregiver input, supplemented by objective sleep–wake data when indicated (43, 45). Subjective measures (e.g., PSQI and sleep diaries) can capture perceived sleep quality, daytime consequences, and nocturnal behaviors, while actigraphy can objectively characterize longer-term sleep–wake patterns in natural environments and help identify circadian rhythm disruption and sleep fragmentation (43, 75). Integrating information from these sources can help identify the predominant sleep problem and its likely contributors. These findings may inform the initial intervention plan (76, 77). Intervention selection should then follow an evidence-informed logic chain. When assessment indicates circadian rhythm sleep–wake disturbance, timed light exposure and routine-based daytime activation may be considered to realign the sleep–wake cycle (78, 79). When insomnia is characterized by conditioned arousal or sleep-related anxiety, CBT-I elements adapted to cognitive level should be selected, with caregiver involvement as needed to support implementation (80, 81). When poor sleep habits or environmental contributors predominate, sleep hygiene education provides a foundational entry point but should be embedded within a broader plan rather than used as a stand-alone strategy (42, 43, 82). Because sleep problems in this population are frequently multifactorial, assessment should also document coexisting contributors (e.g., circadian disruption, inactivity, and nighttime anxiety) so that subsequent care planning can combine components across domains when indicated (83).

### 4.3 Implementation considerations for Non-pharmacological interventions

Implementing NPIs requires balancing expected benefit with feasibility, resources, and setting constraints (84). A stepped-care hierarchy is practical: foundational measures first, structured behavioral/physiological interventions when needed, and combined components when multiple contributors coexist (3, 85). Sleep hygiene education is low cost and broadly applicable, supporting routine stabilization and reducing iatrogenic sleep disruption; however, it is often insufficient as monotherapy for complex neurobehavioral presentations (42, 43, 86). CBT-I is a first-line strategy for chronic insomnia, but in MCI/early dementia it often requires simplification and adaptation to cognitive capacity and may depend on caregiver-facilitated delivery (45, 87, 88). Limited access to trained providers highlights the need for scalable delivery models, including concise caregiver-supported formats and carefully selected digital adaptations (45, 87). Light therapy and exercise may serve as important non-pharmacological intervention options. Timed light exposure is particularly relevant for circadian disruption and requires attention to dose and timing; it may be especially beneficial for individuals with cognitive impairment (43, 49). Institutional settings may facilitate routine delivery, and consistent implementation may reduce sleep fragmentation and daytime sleepiness (43, 49). Exercise interventions in MCI/early dementia are most consistently associated with improvements in subjective sleep quality and total sleep time, whereas effects on sleep efficiency and nocturnal awakenings remain inconsistent across studies (55, 56, 59, 60, 89). Therefore, exercise prescriptions should prioritize feasibility and adherence, tailored to individual preference and physical capacity, rather than pursuing a single optimal modality (42, 49).

Given multifactorial etiology, combined interventions are frequently necessary. Multicomponent protocols integrating morning light exposure, structured daytime activity/exercise, and evening behavioral or relaxation components can address biological, behavioral, and environmental contributors concurrently and may improve sleep efficiency and subjective sleep quality (14, 89). Nevertheless, evidence for the added benefit of multicomponent interventions over single-component approaches remains limited, and the optimal combination of intervention components is unclear (14, 42, 43, 89). Further high-quality trials are needed to clarify the comparative effectiveness of different intervention approaches and combinations. Overall, implementation should be adapted to local resources and care settings, with particular attention to workforce capacity, caregiver support, and feasibility in residential versus home-based care.

### 4.4 Maintaining long-term benefits through monitoring, health education, and care across settings

Because sleep patterns and care needs may evolve as neurodegenerative disease progresses, regular follow-up and individualized support may facilitate ongoing assessment and adaptation of sleep management in individuals with MCI or early dementia (88). Follow-up frequency should be tailored by sleep problem to track symptoms, treatment response, adherence, and safety (43, 45). For acute insomnia, reassessment within 2–4 weeks is recommended (45). For RBD, review at approximately 1 month and every 3–6 months thereafter may be reasonable, with timing individualized to clinical need (44, 45). For chronic insomnia or CRSD, regular follow-up is needed to monitor symptom trajectory, response to non-pharmacological strategies, and adherence over time (45).

Health education and social support may help sustain intervention benefits and should prioritize implementation capacity rather than information alone (90). Providing concise practical materials may facilitate consistent implementation and help reduce variation in sleep-management practices (42–44, 49). Brief rationale explanations (e.g., timing principles for light exposure) and realistic expectation-setting regarding disease progression may improve adherence and reduce caregiver frustration (91). Encouraging bed partners or caregivers to participate in assessment and intervention implementation may support adherence and safety, especially when patient self-management is limited (42–44, 49). In long-term care and community settings, embedding routine non-pharmacological practices (regular schedules, structured daytime light exposure and activities, reduced nighttime noise/light) and providing team-based caregiver training can support the routine implementation of sleep-promoting care (42, 43). Digital and self-help resources may further improve access and adherence (e.g., dCBT-I where appropriate, non-diagnostic self-monitoring, and adjunct relaxation-based strategies), but should be selected for cognitive suitability and used with supervision when needed (45, 49, 50). Overall, maintaining long-term benefit may be supported by coordinated collaboration between clinical teams and caregivers, with follow-up schedules and support strategies adapted to care setting and evolving patient needs (92).

### 4.5 Limitations

Several limitations should be acknowledged. First, the included evidence sources were methodologically heterogeneous and comprised clinical practice guidelines, systematic reviews, expert consensus documents, best practice reports, and evidence summaries. These sources differed in scope, target populations, outcome definitions, methodological approaches, and evidence-grading systems, which limited their direct comparability. Second, synthesizing recommendations across different source types required interpretive judgment when integrating complementary content. Although predefined appraisal and synthesis procedures were applied, the synthesis process may still have involved some degree of subjective judgment. Third, only evidence sources published in English or Chinese were eligible for inclusion, which may have resulted in the omission of relevant evidence published in other languages. Regional and cultural differences in healthcare resources, caregiving arrangements, and clinical practice may also limit the transferability of the synthesized evidence. The evidence should be continuously updated in future research, and appropriate evidence should be selected based on its feasibility, suitability, and effectiveness within specific clinical contexts.

## 5 Conclusions

This study systematically synthesized the best available evidence on non-pharmacological sleep management for sleep disturbances in individuals with MCI and early dementia. The synthesized evidence covers 11 domains. These include risk factors, sleep screening, sleep assessment, sleep hygiene, cognitive behavioral therapy for insomnia, light therapy, exercise, multimodal interventions, other interventions, sleep monitoring, and health education and social support. These evidence statements provide a structured basis for clinical and community professionals to plan and deliver sleep care for this population. The synthesized evidence supports a systematic and individualized approach to non-pharmacological sleep management, with ongoing assessment and collaboration between healthcare professionals and caregivers. Effective implementation may improve quality of life for both patients and caregivers. However, because some of the included evidence was derived from international sources, local adaptation should consider patient preferences, resource availability, and setting-specific constraints.

## Data Availability

The original contributions presented in the study are included in the article/Supplementary Material.
