# Supplementary material for: Summary of best evidence for non-pharmacological management of sleep disturbances in patients with mild cognitive impairment or early dementia: an integrative review
Source: Front Med (Lausanne). 2026 Sep 9;13:1913461. doi: 10.3389/fmed.2026.1913461 (PMC13597332; doi:10.3389/fmed.2026.1913461)
Supplement: Supplementary file 1 [file Datasheet1.docx]

Supplementary Table. 1 The search strategy

**PubMed:**

| **#** | **searches** |
| --- | --- |
| #1 | "Cognitive Dysfunction"[Mesh] OR "Dementia"[Mesh] OR "Alzheimer Disease"[Mesh] |
| #2 | **"Cognitive Dysfunction*"[Title/Abstract] OR Dysfunction*, Cognitive[Title/Abstract] OR Cognitive Impairment*[Title/Abstract] OR Impairment*, Cognitive[Title/Abstract] OR Cognitive Disorder*[Title/Abstract] OR Disorder*, Cognitive[Title/Abstract] OR Mild Cognitive Impairment*[Title/Abstract] OR Cognitive Impairment*, Mild[Title/Abstract] OR Impairment*, Mild Cognitive[Title/Abstract] OR Cognitive Decline*[Title/Abstract] OR Decline*, Cognitive[Title/Abstract] OR Mental Deterioration*[Title/Abstract] OR Deterioration*, Mental[Title/Abstract] OR** dementia*[Title/Abstract] OR alzheimer*[Title/Abstract] |
| #3 | #1 OR #2 |
| #4 | "**Dyssomnias"**[MeSH Terms] |
| #5 | **("sleep"[Title/Abstract] OR "sleep disorder"[Title/Abstract] OR "sleep problems"[Title/Abstract] OR**  **"sleep disturbances"[Title/Abstract] OR "sleep quality"[Title/Abstract] OR "insomnia"[Title/Abstract] OR**  **"sleepiness"[Title/Abstract] OR "somnolence"[Title/Abstract] OR "sleep apnea"[Title/Abstract] OR**  **"snoring"[Title/Abstract] OR "sleep disordered breathing"[Title/Abstract] OR**  **"obstructive sleep apnea"[Title/Abstract] OR "restless legs syndrome"[Title/Abstract] OR**  **"periodic limb movements disorder"[Title/Abstract] OR "REM sleep behavior disorder"[Title/Abstract] OR**  **"sleep movement disorder"[Title/Abstract] OR "parasomnias"[Title/Abstract] OR**  **"sleep-wake disruption"[Title/Abstract] OR "circadian rhythm sleep disorder"[Title/Abstract] OR**  **"sleep phase"[Title/Abstract] OR "sleep pattern"[Title/Abstract])** |
| #6 | #4 OR #5 |
| #7 | ("guideline"[Title/Abstract] OR "consensus"[Title/Abstract] OR "systematic review"[Title/Abstract] OR "meta-analysis"[Title/Abstract] OR "evidence summary"[Title/Abstract] OR "recommendation"[Title/Abstract] OR "clinical decision"[Title/Abstract] OR "best practice"[Title/Abstract]) |
| #8 | #3 AND #6 AND #8 |

**Web of science:**

| **#** | **searches** |
| --- | --- |
| #1 | TS=("Cognitive Dysfunction*" OR "Cognitive Impairment*" OR "Cognitive Disorder*" OR "Mild Cognitive Impairment*" OR "Cognitive Decline*" OR "Mental Deterioration*"OR dementia* OR alzheimer*) |
| 2 | **TS=("sleep" OR "sleep disorder" OR "sleep problems" OR "sleep disturbances" OR "sleep quality" OR**  **"insomnia" OR "sleepiness" OR "somnolence" OR "sleep apnea" OR "snoring" OR "sleep disordered breathing" OR**  **"obstructive sleep apnea" OR "restless legs syndrome" OR "periodic limb movement disorder" OR**  **"REM sleep behavior disorder" OR "sleep movement disorder" OR "parasomnias" OR**  **"sleep-wake disruption" OR "circadian rhythm sleep disorder" OR "sleep phase" OR "sleep pattern")** |
|  | **TS=**("guideline" OR "consensus" OR "systematic review" OR "meta-analysis" OR "evidence summary" OR "recommendation" OR "clinical decision" OR "best practice") |
| 3 | #1 AND #2 AND #3 |

**Cochrane library:**

| **#** | **searches** |
| --- | --- |
| 1 | MeSH descriptor: [Cognitive Dysfunction] explode all trees |
| 2 | MeSH descriptor: [Dementia] explode all trees |
| 3 | (Cognitive Dysfunction* OR Cognitive Impairment* OR Cognitive Disorder* OR Mild Cognitive Impairment* OR Cognitive Decline* OR Mental Deterioration*OR dementia* OR alzheimer*):ti,ab,kw |
| 4 | #1 OR #2 OR #3 |
| 5 | MeSH descriptor: [Dyssomnias] explode all trees |
| 6 | (sleep OR sleep disorder OR sleep problems OR sleep disturbances OR sleep quality OR insomnia OR sleepiness OR somnolence OR sleep apnea OR snoring OR sleep disordered breathing OR obstructive sleep apnea OR restless legs syndrome OR periodic limb movement disorder OR REM sleep behavior disorder OR sleep movement disorder OR parasomnias OR sleep-wake disruption OR circadian rhythm sleep disorder OR sleep phase OR sleep pattern):ti,ab,kw |
| 7 | #6 OR #5 |
| 8 | (guideline OR consensus OR systematic review OR meta-analysis OR evidence summary OR recommendation OR best practice OR clinical decision):ti,ab,kw |
| 9 | #4 AND # AND #8 |

**CINAHL**

| 1 | SU "Cognitive Dysfunction*" OR SU "Dysfunction*, Cognitive" OR SU "Cognitive Impairment*" OR SU "Impairment*, Cognitive" OR SU "Cognitive Disorder*" OR SU "Disorder*, Cognitive" OR SU "Mild Cognitive Impairment*" OR SU "Cognitive Impairment*, Mild" OR SU "Impairment*, Mild Cognitive" OR SU "Cognitive Decline*" OR SU "Decline*, Cognitive" OR SU "Mental Deterioration*" OR SU "Deterioration*, Mental" OR SU "dementia*" OR SU "alzheimer*" |
| --- | --- |
| 2 | SU "sleep" OR SU "sleep disorder" OR SU "sleep problems" OR SU "sleep disturbances" OR SU "sleep quality" OR SU "insomnia" OR SU "sleepiness" OR SU "somnolence" OR SU "sleep apnea" OR SU "snoring" OR SU "sleep disordered breathing" OR SU "obstructive sleep apnea" OR SU "restless legs syndrome" OR SU "periodic limb movements disorder" OR SU "REM sleep behavior disorder" OR SU "sleep movement disorder" OR SU "parasomnias" OR SU "sleep-wake disruption" OR SU "circadian rhythm sleep disorder" OR SU "sleep phase" OR SU "sleep pattern" |
| 3 | SU "guideline" OR SU "consensus" OR SU "systematic review" OR SU "meta-analysis" OR SU "evidence summary" OR SU "recommendation" OR SU "clinical decision" OR SU "best practice" |
| 4 | #1 AND #2 AND #3 |

**Embase:**

| **#** | **searches** |
| --- | --- |
| 1 | 'cognitive defect'/exp OR 'alzheimer disease'/exp OR 'dementia'/exp |
| 2 | 'cognitive dysfunction*':ab,ti OR 'cognitive impairment*':ab,ti OR 'cognitive disorder*':ab,ti OR 'mild cognitive impairment*':ab,ti OR 'cognitive decline*':ab,ti OR 'mental deterioration*':ab,ti OR 'dementia*':ab,ti OR 'alzheimer*':ab,ti OR |
| 3 | #1 OR #2 |
| 4 | 'dyssomnia'/exp |
| 5 | "sleep":ab,ti OR "sleep disorder":ab,ti OR "sleep problems":ab,ti OR "sleep disturbances":ab,ti OR "sleep quality":ab,ti OR "insomnia":ab,ti OR "sleepiness":ab,ti OR "somnolence":ab,ti OR "sleep apnea":ab,ti OR "snoring":ab,ti OR "sleep disordered breathing":ab,ti OR "obstructive sleep apnea":ab,ti OR "restless legs syndrome":ab,ti OR  "periodic limb movement disorder":ab,ti OR "REM sleep behavior disorder":ab,ti OR "sleep movement disorder":ab,ti OR "parasomnias":ab,ti OR "sleep-wake disruption":ab,ti OR "circadian rhythm sleep disorder":ab,ti OR "sleep phase":ab,ti OR "sleep pattern":ab,ti |
| 6 | #4 OR #5 |
| 7 | 'practice guideline'/exp OR 'meta analysis'/exp OR 'systematic review'/exp |
| 8 | "guideline":ab,ti OR "consensus*":ab,ti OR  "systematic review":ab,ti OR "meta-analysis":ab,ti OR  "evidence summary":ab,ti OR "recommendation":ab,ti OR "best practice":ab,ti OR "clinical decision":ab,ti |
| 9 | #7 OR #8 |
| 10 | #3 AND #6 AND #9 |

**CBM:**

| **#** | **searches** |
| --- | --- |
| 1 | "认知障碍"[不加权:扩展] |
| 2 | "轻度认知障碍"[常用字段:智能] OR "认知损害"[常用字段:智能] OR "精神衰退"[常用字段:智能] OR "认知减退"[常用字段:智能] OR "轻度神经认知障碍"[常用字段:智能] OR "阿尔兹海默症"[常用字段:智能] OR "痴呆"[常用字段:智能] |
| 3 | (#2) OR (#1) |
| 4 | "睡眠障碍"[常用字段:智能] OR "睡眠质量"[常用字段:智能] OR "睡眠觉醒障碍"[常用字段:智能] OR "睡眠障碍昼夜节律"[常用字段:智能] OR "睡眠不足"[常用字段:智能] OR "时间生物学障碍"[常用字段:智能] OR "睡眠"[常用字段:智能] OR "失眠"[常用字段:智能] OR "睡眠紊乱"[常用字段:智能] OR "睡眠问题"[常用字段:智能] |
| 5 | "指南"[常用字段:智能] OR "共识"[常用字段:智能] OR  "系统评价"[常用字段:智能] OR "Meta分析"[常用字段:智能] OR "Meta整合"[常用字段:智能] OR "证据总结"[常用字段:智能] OR "最佳实践"[常用字段:智能] OR "临床决策"[常用字段:智能] |
| 6 | (#5) AND (#4) AND (#3) |

**CNKI**

| **#** | **searches** |
| --- | --- |
| 1 | SU=认知障碍+轻度认知障碍+认知损害+精神衰退+认知减退+轻度神经认知障碍+痴呆+阿尔茨海默病 |
| 2 | SU=睡眠障碍+睡眠质量+睡眠觉醒障碍+睡眠障碍昼夜节律+睡眠不足+时间生物学障碍+睡眠+失眠+睡眠紊乱+睡眠问题 |
| 3 | SU=指南+专家共识+系统评价+Meta分析+Meta整合+证据总结+最佳实践+临床决策 |
| 4 | #1 AND #2 AND #3 |

**Wangfang**

| **#** | **searches** |
| --- | --- |
| 1 | 主题:(睡眠障碍 OR 睡眠质量 OR 睡眠觉醒障碍 OR 睡眠障碍昼夜节律 OR 睡眠不足 OR 时间生物学障碍 OR 睡眠 OR 失眠 OR 睡眠紊乱 OR 睡眠问题)  AND  主题:(指南 OR 专家共识 OR 系统评价 OR Meta分析 OR Meta整合 OR 证据总结 OR 最佳实践 OR 临床决策)  AND  主题:(认知障碍 OR 认知损害 OR 精神衰退 OR 认知减退 OR 轻度神经认知障碍 OR 痴呆 OR 阿尔茨海默病) |

Supplementary Table 2: Methodological quality evaluation results of the guidelines (n =2)

| Included literature | Standardised scores in various domains (%) | | | | | | ≥ 60% field number  (n) | ≥ 30% field number  (n) | Recommendation level |
| --- | --- | --- | --- | --- | --- | --- | --- | --- | --- |
|  | Scopes and objects | Participant | Rigour of the guidelines | Clarity of guidelines | Application of guidelines | Independence of the guide |  |  |  |
| B.Guarnieri • M | 94.44 | 61.11 | 60.42 | 94.44 | 58.33 | 16.67 | 4 | 5 | B |
| R Robert Auger | 97.22 | 83.33 | 82.29 | 91.67 | 62.5 | 83.33 | 6 | 6 | A |

Supplementary Table 3: Quality evaluation results of the clinical decisions and best practice reports (n =7 )

|  | Evaluation Items | | | | | | | | | |
| --- | --- | --- | --- | --- | --- | --- | --- | --- | --- | --- |
| Evidence summary  and best Practice | ① | ② | ③ | ④ | ⑤ | ⑥ | ⑦ | ⑧ | ⑨ | ⑩ |
| Larson, EB | Yes | Yes | Yes | No | Partially Yes | Yes | Yes | Yes | Yes | Yes |
| Feinsilver, SH. | Yes | Yes | Yes | No | Partially Yes | Yes | Yes | Yes | Yes | Yes |
| Ofer Jacobowitz | Yes | Yes | Yes | No | Yes | Yes | Yes | Yes | Yes | Yes |
| Joshua Hyong-Jin Cho | Yes | Yes | Yes | No | Yes | Yes | Yes | Yes | Yes | Yes |
| Fariba KA, Tadi P | Yes | Yes | Yes | No | Partially Yes | Yes | Yes | Yes | Yes | Yes |
| Petersen, RC | Yes | Yes | Yes | No | Partially Yes | Yes | Yes | Yes | Yes | Yes |
| Petersen, RC | Yes | Yes | Yes | No | Partially Yes | Yes | Yes | Yes | Yes | Yes |

Note:①Is the summary specific in scope and application ?② Is the authorship of the summary transparent? ③Are the reviewer (s)/ editor (s) of the summary transparent ? ④Are the search methods transparent and comprehensive? ⑤ Is the evidence grading system transparent and translatable? ⑥ Are the recommendations clear? ⑦Are the recommendations appropriately cited？⑧Are the recommendations current ? ⑨Is the summary unbiased ?⑩Can this summary be applied to your patient (s)?

Supplementary Table 4: Quality evaluation results of expert consensuses (n =4)

| Expert consensus | Evaluation entry | | | | | | Overall quality |
| --- | --- | --- | --- | --- | --- | --- | --- |
|  | ① | ② | ③ | ④ | ⑤ | ⑥ |  |
| Manuel Montero-Odasso | Yes | Yes | Yes | Yes | Yes | Yes | Include |
| Chinese Sleep Research Society | Yes | Yes | Yes | Yes | Yes | Yes | Include |
| Chinese Sleep Research Society | Yes | Yes | Yes | Yes | Yes | Yes | Include |
| Wang et al. | Yes | Yes | Yes | Yes | Yes | Yes | Include |

Note: ① Is the source of the opinion clearly stated? ② Are the opinions from influential experts in the field? ③Are the opinions presented centered on the interests of the people involved in the study? ④Is the stated conclusion based on the results of the analysis? Are opinions expressed logically? ⑤ Whether to refer to other existing literature? ⑥ Are there any inconsistencies between the opinions presented and the previous literature?

Supplementary Table 5: Quality evaluation results of included systematic reviews (n =9)

| Included literature | Evaluation entry | | | | | | | | | | | | | | | |
| --- | --- | --- | --- | --- | --- | --- | --- | --- | --- | --- | --- | --- | --- | --- | --- | --- |
|  | Q1 | Q2 | Q3 | Q4 | Q5 | Q6 | Q7 | Q8 | Q9 | Q10 | Q11 | Q12 | Q13 | Q14 | Q15 | Q16 |
| Jeff W Jin | Y | N | Y | Y | Y | Y | Y | Y | Y | N | No meta-analysis conducted | No meta-analysis conducted | Y | Y | No meta-analysis conducted | Y |
| Gao et al. | Y | Y | Y | Y | Y | Y | Y | Y | Y | N | Y | Y | Y | Y | Y | N |
| Huei-Ling Chiu | Y | Y | Y | Y | Y | Y | Y | Y | Y | N | Y | Y | Y | Y | Y | Y |
| Junlei Zhang | Y | Y | Y | Y | Y | Y | Y | Y | Y | N | Y | Y | Y | Y | Y | Y |
| Ahn, J, | Y | Y | Y | Y | Y | Y | Y | Y | Y | N | Y | Y | Y | Y | Y | Y |
| Chen, L | Y | Y | Y | Y | Y | Y | Y | Y | Y | N | Y | Y | Y | Y | Y | Y |
| Liu, X, | Y | Y | Y | Y | Y | Y | Y | Y | Y | N | Y | Y | Y | Y | Y | Y |
| Rónán O'Caoimh | Y | Y | Y | Y | Y | Y | Y | Y | Y | N | Y | Y | Y | Y | Y | Y |
| Jonathan Blackman | Y | Y | Y | Y | Y | Y | Y | Y | Y | N | No meta-analysis conducted | No meta-analysis conducted | Y | Y | No meta-analysis conducted | Y |

Note: Y: Yes; N: No; Q1: Did the research questions and inclusion criteria for the review include the components of PICO? Q2: Did the report of the review contain an explicit statement that the review methods were established prior to the conduct of the review and did the report justify any significant deviations from the protocol? Q3: Did the review authors explain their selection of the study designs for inclusion in the review? Q4: Did the review authors use a comprehensive literature search strategy? Q5: Did the review authors perform study selection in duplicate? Q6: Did the review authors perform data extraction in duplicate? Q7: Did the review authors provide a list of excluded studies and justify the exclusions? Q8: Did the review authors describe the included studies in adequate detail? Q9: Did the review authors use a satisfactory technique for assessing the risk of bias (RoB) in individual studies that were included in the review? Q10: Did the review authors report on the sources of funding for the studies included in the review? Q11: If meta-analysis was performed, did the review authors use appropriate methods for statistical combination of results? Q12: If meta-analysis was performed, did the review authors assess the potential impact of RoB in individual studies on the results of the meta-analysis or other evidence synthesis? Q13: Did the review authors account for RoB in primary studies when interpreting/discussing the results of the review? Q14: Did the review authors provide a satisfactory explanation for, and discussion of, any heterogeneity observed in the results of the review? Q15: If they performed quantitative synthesis did the review authors carry out adequate investigation of publication bias (small study bias) and discuss its likely impact on the results of the review? Q16: Did the review authors report any potential sources of conflict of interest, including any funding they received for conducting the review?
